# *Bacillus subtilis* DES-59 Modulates Microbial Community Structure and Aroma Formation in Cigar Fermentation to Improve the Quality of Heat-Not-Burn (HnB) Tobacco

**DOI:** 10.4014/jmb.2605.05012

**Published:** 2026-09-02

**Authors:** Lu-Xin Liang, Ming-Jun Zhu, Lu Zhao

**Affiliations:** 1 School of Biology and Biological Engineering, Guangdong Key Laboratory of Fermentation and Enzyme Engineering, South China University of Technology, Guangzhou Higher Education Mega Center, Panyu, Guangzhou 510006, P. R. China; 2 Yunnan Academy of Tobacco Agricultural Sciences, Kunming 650021, P. R. China; 3 China Tobacco Breeding Technology Innovation Center, Kunming 650021, P. R. China; 4 Yunnan Daguan Laboratory, Kunming 650021, P. R. China

**Keywords:** *Bacillus subtilis* DES-59, Cigar, Fermentation, HnB cigarettes, Sensory quality, Microbial community

## Abstract

Cigar tobacco is characterized by high protein and low sugar contents, which limits the sensory performance of cigar-flavored heat-not-burn (HnB) products. In this study, Yunxue 6 cigar tobacco powder was fermented with *Bacillus subtilis* DES-59 for 7 days under solid-state culture at 25°C and 65% relative humidity. Compared with the control group, fermented tobacco exhibited a 10.55% reduction in protein, alongside 30.10% and 47.86% increases in total free amino acids and reducing sugars, respectively. Py-GC/MS analysis at 350°C showed increased release of pyrolytic aroma compounds, with the relative abundances of esters, aldehydes / ketones, and phenols increasing by 86.95%, 58.53%, and 35.69%, respectively. Sensory evaluation of HnB cigarettes manufactured from the fermented tobacco powder showed an overall sensory score improvement from 79.00 to 83.00, mainly driven by enhanced aroma and flavor. High-throughput sequencing demonstrated that inoculation reshaped the bacterial community, increasing the relative abundance of *Bacillus* from 12.20% to 52.40%. PICRUSt functional prediction suggested an increased functional potential for amino acid and carbohydrate metabolism in the treated group. Overall, inoculation with *B. subtilis* DES-59 was associated with changes in the chemical composition, bacterial community structure, pyrolysis-derived aroma profile, and sensory characteristics of cigar tobacco powder. These findings provide preliminary evidence supporting the potential application of *B. subtilis* DES-59 in the fermentation of cigar tobacco powder for the development of cigar-flavored raw materials for HnB products.

## Introduction

With the continuous development of global tobacco control policies and public health awareness, heat-not-burn (HnB) cigarettes represent a novel class of nicotine delivery systems, which are fundamentally different from conventional cigarettes relying on high-temperature combustion above 900°C [1-3]. Differently, HnB products generate and release nicotine and flavor compounds in the form of aerosols via heating at a lower temperature range of 300–350°C. Compared with high-temperature combustion, this lower operating temperature is associated with reduced thermal decomposition of tobacco constituents. Accordingly, existing studies have reported lower emissions of harmful components including tar, carbon monoxide and polycyclic aromatic hydrocarbons (PAHs) in HnB aerosols relative to conventional cigarette smoke [3, 4]. Despite this distinct heating characteristic, HnB products still face notable technical limitations. Limited by relatively low heating temperature, most HnB products exhibit low aroma release efficiency and weak smoke flavor intensity, which hardly satisfy sensory demands from consumers [5, 6]. Therefore, optimizing the sensory properties of HnB products via raw material modification has drawn increasing attention in both academic research and the tobacco industry [7-9].

Cigar tobacco possesses rich flavor layers and distinctive volatile aromatic components, which can effectively enrich the flavor profile of tobacco products and serve as a raw material option to compensate for the insufficient aroma of HnB cigarettes [10]. However, cigar tobacco is characterized by high nitrogen and low sugar chemical features, containing abundant macromolecules such as protein and cellulose, while the contents of total soluble sugars and reducing sugars are generally low [11, 12]. During the heating process, these macromolecules are susceptible to thermal pyrolysis and produce irritative substances, bitter taste and undesirable off-flavors, which deteriorate smoke smoothness and smoking comfort [8, 13-15]. Excessive protein accumulation in tobacco leaves tends to generate pungent, sour and peculiar off-flavors during thermal heating, thereby impairing the overall sensory experience[8]. In contrast, endogenous small-molecule sugars and amino acids in tobacco act as crucial flavor precursors, which endow tobacco with rich, mellow and harmonious aroma attributes and are essential for improving the sensory quality of cigar-flavored HnB cigarettes. Accordingly, maintaining the inherent flavor characteristics of cigar tobacco while mitigating the adverse sensory effects derived from macromolecular substances remains a key challenge for developing high-quality cigar-type HnB products.

Fermentation is a classic and effective approach to improve tobacco quality. Microbial metabolism and enzymatic catalysis can efficiently degrade macromolecular compounds and synthesize flavor precursors, thereby optimizing the chemical composition and sensory quality of tobacco [16-20]. Nevertheless, natural tobacco fermentation usually requires a long production cycle of 1–3 years and presents unstable quality, which cannot meet the large-scale and standardized production requirements of modern tobacco industry [21, 22]. Moreover, indigenous microorganisms from tobacco leaves and surrounding environments easily cause mildew contamination, microbial community dysbiosis and flavor deterioration, which greatly restrict the industrial application of natural fermentation [23]. In this context, inoculation fermentation with functional microorganisms has become a promising targeted strategy for tobacco quality regulation [24]. Among multiple functional strains, aroma-producing microorganisms including *Bacillus*, yeasts and *Acinetobacter* can degrade macromolecular substances and synthesize favorable volatile components, thereby effectively improving tobacco aroma and sensory performance [7, 25-29]. In particular, *Bacillus subtilis* has attracted extensive attention owing to its excellent enzyme secretion ability and high biological safety [24, 30]. Existing studies have confirmed that *B. subtilis* plays multiple regulatory roles in tobacco fermentation. It can secrete various extracellular hydrolases including protease, cellulase and amylase to degrade tobacco macromolecules and reduce smoke irritation and undesirable odors [24, 31]. Meanwhile, microbial metabolism promotes the accumulation of flavor precursors such as amino acids and reducing sugars. These precursors further participate in the Maillard reaction to form heterocyclic aroma compounds including pyrazines and furans [32, 33]. For instance, Dai *et al*. screened a thermophilic *B. subtilis* ZIM3 with high amylase and cellulase activities to decompose starch and cellulose in tobacco efficiently [34]; Huang *et al*. reported that *B. subtilis* H11 reshaped the epiphytic microbial community and facilitated the synthesis of key aroma compounds [35]; Ning *et al*. also confirmed that *B. subtilis* B1 improved the sensory quality of HnB cigarettes during tobacco powder solid-state fermentation [24]. These findings demonstrate broad application potential of *B. subtilis* in tobacco fermentation.

However, most previous studies have focused on the microbial fermentation of flue-cured tobacco for conventional combustible cigarettes, whereas relatively few have investigated the fermentation of cigar tobacco powder for HnB applications. To the best of our knowledge, the application of *B. subtilis* DES-59 in the fermentation of cigar tobacco powder has not been reported. Furthermore, conventional volatile analysis performed at ambient temperature does not adequately reflect the aroma release characteristics under the actual heating conditions of HnB products.

To address these research gaps, the present study investigated the solid-state fermentation of Yunxue 6 cigar tobacco powder using *B. subtilis* DES-59. The larger specific surface area of tobacco powder facilitates microbial colonization and is compatible with the production process of reconstituted tobacco for HnB products. Py-GC/MS analysis at 350°C was employed to simulate the typical heating conditions of commercial HnB products for aroma evaluation. Changes in chemical composition, pyrolysis-derived aroma compounds, sensory characteristics, and bacterial community structure were systematically analyzed to assess the effects of *B. subtilis* DES-59 fermentation on cigar tobacco powder. The findings provide preliminary evidence supporting the potential application of *B. subtilis* DES-59 in the development of cigar-flavored raw materials for HnB products.

## Materials and Methods

### Materials

Middle leaves of Yunxue 6 cigar tobacco were provided by the Tobacco Research Institute of Yunnan Academy of Agricultural Sciences. After air-curing and preliminary agricultural fermentation, the tobacco stems were removed, and the leaves were ground and sieved through a 200-mesh sieve to obtain cigar tobacco powder. The sealed cigar tobacco powder used for fermentation had an initial moisture content of approximately 9.1%. Tobacco powder rather than intact tobacco leaves was selected, as it is more suitable for the preparation of tobacco sheets and heat-not-burn products. Meanwhile, the larger specific surface area of tobacco powder facilitates microbial attachment, strain colonization and nutrient mass transfer, thereby enhancing fermentation performance and chemical component transformation efficiency [24].

*B. subtilis* DES-59 was isolated, purified and preserved by the Fermentation Engineering Laboratory of the School of Biology and Biological Engineering, South China University of Technology. *B. subtilis* DES-59 is a high-yield strain capable of secreting extracellular neutral protease.

## Methods

### Cigar Tobacco Fermentation Inoculated with *B. subtilis* DES-59

The specific operation steps are as follows. (1) Strain cultivation. The activated DES-59 was inoculated into Luria-Bertani (LB) liquid medium (10 g/L tryptone, 5 g/L yeast extract, 10 g/L sodium chloride, pH adjusted to 7.0, autoclaved at 115°C for 30 min), and cultured with shaking in a shaker at 25°C and 220 rpm for 12 h to obtain the seed culture. (2) Preparation of fermentation inoculum. According to the inoculation amount of 2% (v/v), the above seed culture was transferred to freshly prepared LB liquid medium, and cultured with shaking for another 12 h under 25°C and 220 rpm. After cultivation, the bacterial liquid was centrifuged at 3000 rpm for 5 min, the supernatant was discarded, and an equal volume of sterile distilled water was added to resuspend the bacterial cells to prepare a bacterial suspension with a concentration of approximately 1 × 10^9^ CFU/mL, which was used as the final fermentation inoculum. The concentration of the bacterial suspension was determined by the plate counting method on LB solid medium. (3) Solid-state fermentation of cigar tobacco. Accurately weigh 20 g of cigar tobacco powder and place it in a 350 mL tissue culture flask. *B. subtilis* DES-59 treatment (BST) group setting: The prepared fermentation inoculum was uniformly sprayed onto the cigar tobacco powder at a rate of 0.6 mL g^-1^ to adjust the initial moisture content of all samples to 30.0%. The samples were then thoroughly mixed with a sterile glass rod to ensure even distribution of the inoculum throughout the tobacco powder. Place the inoculated samples in a constant temperature and humidity incubator, set the typical culture conditions to 25°C and 65% relative humidity [36], and conduct solid-state fermentation for 7 days [24]. During fermentation, the samples were manually turned over with a sterile glass rod every 24 h to ensure uniform fermentation. The control group (Control) was treated with an equal volume of sterile distilled water instead of the fermentation inoculum, and all other operation conditions were consistent with the BST group. All experiments were performed in triplicate. The moisture content (Table S1) and viable microorganisms (Table S2) at the initial and final fermentation stages are provided in supplementary materials.

### Determination Methods of Chemical Composition

Cigar tobacco powder samples at Day 0 and Day 7 were inactivated at 100°C for 30 min, dried at 50°C to constant weight, ground with a grinder, sieved through a 200-mesh sieve, and then dried at 100°C for 2 h to constant weight for chemical component detection. The contents of total soluble sugars and reducing sugars, total free amino acids, cellulose, protein, and total alkaloids were determined in accordance with the standards YC/T 159-2019, GB/T 8314-2013, GB 5009.88-2014, YC/T 249-2008, and YC/T 468-2013, respectively. All the above indicators were determined in triplicate.

### Determination Method of Aroma Components in Pyrolysis Smoke

A 0.4 g sample of fermented (Day 7) cigar tobacco powder was weighed and placed into a quartz tube of a rapid heating furnace. A 20% O_2_-80% N_2_ mixed gas was introduced at 0.2 L/min via a flow control system; after 3 min of ventilation, the furnace was heated from room temperature to 350°C within 20 s (computer-controlled) and held for 5 min, with smoke particulate phase trapped by a Cambridge filter. After heating, the furnace lid was opened for 5 min of cooling before removing the filter. The used filter was folded into an Erlenmeyer flask, added with 60 mL dichloromethane, sealed, and ultrasonically extracted at room temperature for 30 min to obtain the aroma extract. The extract was mixed with internal standard, concentrated, filtered through a 0.45 μm organic membrane into a chromatographic vial. The extract was analyzed by GC/MS following the method reported by Zhang *et al*. [22], and detailed descriptions of the analytical method have been supplemented in Supplementary Method S1.

### Evaluation Method of Sensory Quality

Fermented cigar tobacco powder collected on Day 7 was inactivated in an oven at 100°C for 30 min, followed by drying at 40°C to a constant weight. The treated tobacco was manufactured into cigar-flavored HnB cigarettes in accordance with factory standards. The evaluation method and scoring standards referred to Q/YNYZ.J04.022-2015. An internal core heating cigarette device was adopted in this evaluation, and the maximum heating temperature of the device temperature rise curve was approximately 350°C. All sensory quality evaluation was organized by the Technology Center of Shandong China Tobacco Industry Co., Ltd., with a panel of 5 professional assessors participating. Prior to the formal evaluation, all panelists underwent standardized training, including flavor recognition, scale interpretation, and scoring consistency calibration, to establish uniform evaluation criteria. All samples were assigned random anonymous codes to conceal treatment identities, and the presentation order was fully randomized for each panelist to minimize potential evaluation bias. The sensory evaluation contained 6 core indicators, aroma and flavor (30 points), taste (25 points), irritation (15 points), smoke volume (10 points), physiological strength (10 points), harmony (10 points), with a total score of 100 points. Detailed scoring standards are shown in Table S3. The final score was calculated as the average value of independent scores provided by the 5 panelists.

### Determination Method of Microbial Community

Genomic DNA was extracted from the cigar tobacco powder samples after 7 days of fermentation. The V3-V4 region of the bacterial 16S rRNA gene was amplified using the specific primers 341F and 806R, followed by high-throughput sequencing on the Novaseq-PE250 platform. QIIME 2 software was employed for species annotation, normalization, and diversity analysis. Specifically, alpha and beta diversity analyses were conducted based on the normalized sequencing data. All DNA extraction, amplicon sequencing, and related bioinformatics analyses were performed by Beijing Novogene Technology Co., Ltd.,(China).

### Data Analysis Methods

Experimental data were sorted using Excel 2021. Statistical analysis was performed with SPSS 27.0.1 software, and one-way analysis of variance (ANOVA) combined with Duncan's multiple comparison test was used to determine the significant differences among groups, with the significance level set at *p* < 0.05. Graphs and tables were plotted using Excel 2021 and Origin 2021.

## Results and Discussion

### Changes in Chemical Compositions of Cigar Tobacco Powder

Chemical composition is the fundamental determinant of tobacco quality, directly affecting its pyrolysis behavior and sensory properties [37, 38]. Besides cultivar selection and agronomic optimization, microbial fermentation is one of the most effective strategies to improve tobacco quality by reshaping its chemical profile [39]. *B. subtilis* is widely recognized as a promising microbial agent for tobacco fermentation due to its strong capacity to secrete extracellular hydrolases. For instance, Dai *et al*. isolated a thermophilic *B. subtilis* ZIM3 from aged flue-cured tobacco, which exhibited high amylase and cellulase activities and efficiently degraded starch and cellulose in tobacco leaves [34]. Ye *et al*. further reported that *B. subtilis* FYZ1-3, isolated from tobacco waste, not only degraded starch and protein but also showed high nicotine tolerance and inhibited common tobacco pathogenic fungi [40].

**Fig. 1:**
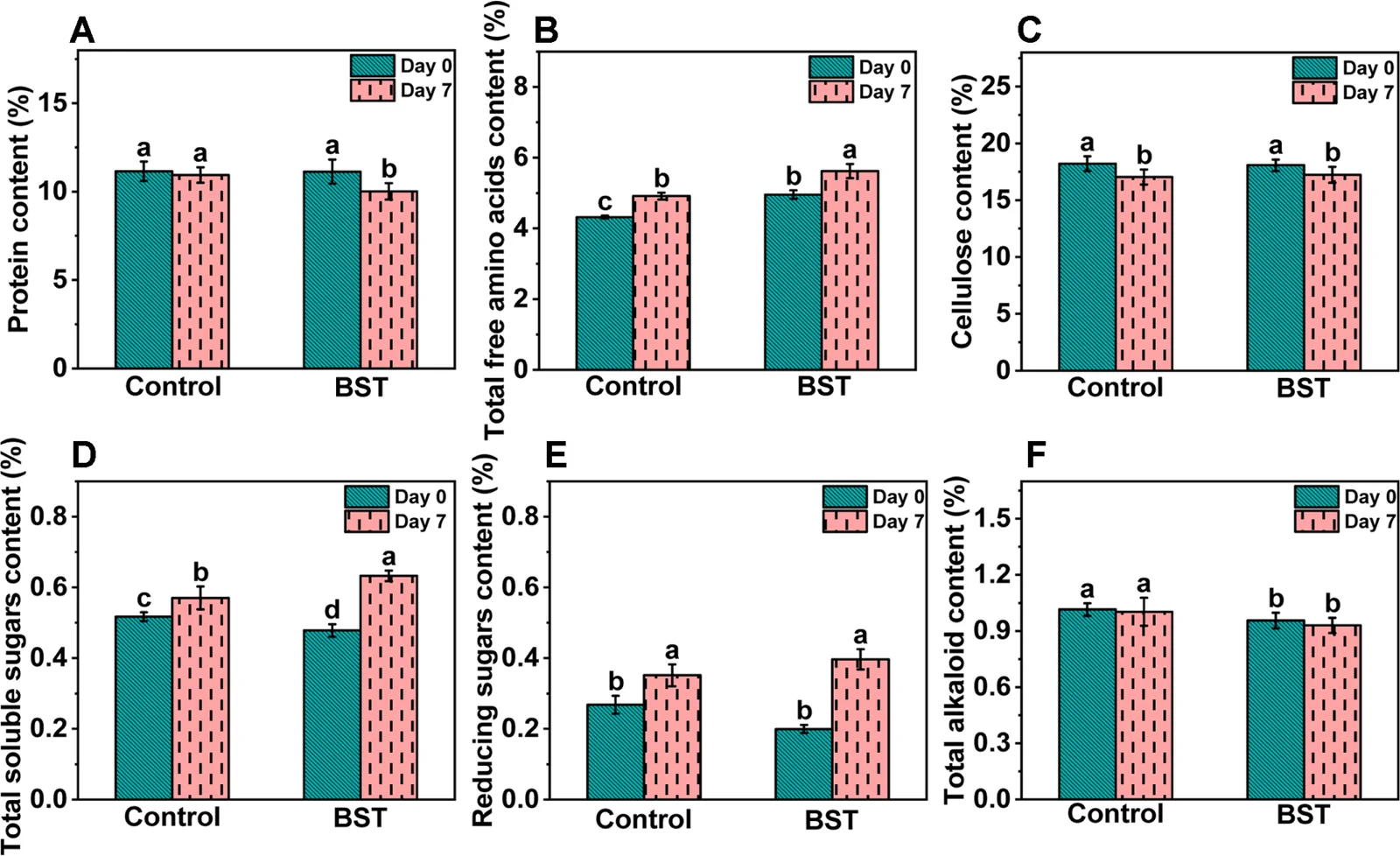
Changes in chemical compositions of cigar tobacco powder before and after fermentation. (**A**) Protein content; (**B**) Total free amino acids content; (**C**) Cellulose content; (**D**) Total soluble sugars content; (**E**) Reducing sugars content; (**F**) Total alkaloid content; Control: control group; BST: *Bacillus subtilis* DES-59 group. Data are presented as mean value ± standard deviation (SD, n = 3). Different lowercase letters indicate significant differences among groups at *p* < 0.05 according to Duncan’s multiple range test.

In this study, after 7 days of solid-state fermentation, the protein content in the BST group was significantly reduced compared with its initial level (Day 0) and with the control group at Day 7 (*p* < 0.05, Fig. 1A). Specifically, the protein content of the BST group decreased by 10.55%. This reduction may contribute to changes in nitrogen-containing pyrolysis products during heating and could potentially influence the sensory characteristics of HnB aerosol, such as smoothness [15]. Consistent with the reduced protein abundance, the total free amino acids content of the BST group increased by 30.10% relative to Day 0 (Fig. 1B). Although the control group also showed a moderate increase in amino acids driven by native microbial activity, the increase in the BST group was notably higher, suggesting that *B. subtilis* DES-59 fermentation further promoted the accumulation of amino acids, which are key precursors for the Maillard reaction and aroma formation [14, 41]. Cellulose contents in both groups decreased significantly after fermentation (*p* < 0.05, Fig. 1C), suggesting that microbial activity played a crucial role in promoting cellulose degradation. The BST group also exhibited significantly higher contents of total soluble sugars and reducing sugars than the control group at Day 7, with increases of 22.36% and 47.86%, respectively (Fig. 1D and 1E). These sugars may act as vital substrates for the Maillard reaction and caramelization, which may contribute to the production of sweet, mellow aroma substances and the sweet taste of HnB aerosol [14]. Notably, reducing sugars accumulation was more pronounced in the BST group, implying that microbial metabolism promoted the hydrolysis of polysaccharides into reducing sugars, further potentially optimizing the sugar/protein ratio and the flavor precursor balance. Total alkaloid content showed a slight decrease in the BST group after fermentation (Fig. 1F). A moderate reduction in alkaloids may alleviate smoke harshness and bitterness without compromising the physiological satisfaction of HnB cigarettes, thus potentially contributing to a smoother smoking experience [42, 43].

In summary, fermentation with *B. subtilis* DES-59 was associated with changes in the chemical composition of cigar tobacco powder. These changes included decreased protein content, increased levels of free amino acids and reducing sugars, and a moderate reduction in alkaloid concentrations, suggesting that DES-59 fermentation may influence the distribution of key chemical components related to tobacco quality. These changes may collectively shift the chemical profile of cigar tobacco toward a more balanced state, which could provide a beneficial material basis for improving the sensory quality of cigar-flavored HnB products.

### Aroma Compounds Released from the Pyrolysis of Cigar Tobacco Powder

**Fig. 2:**
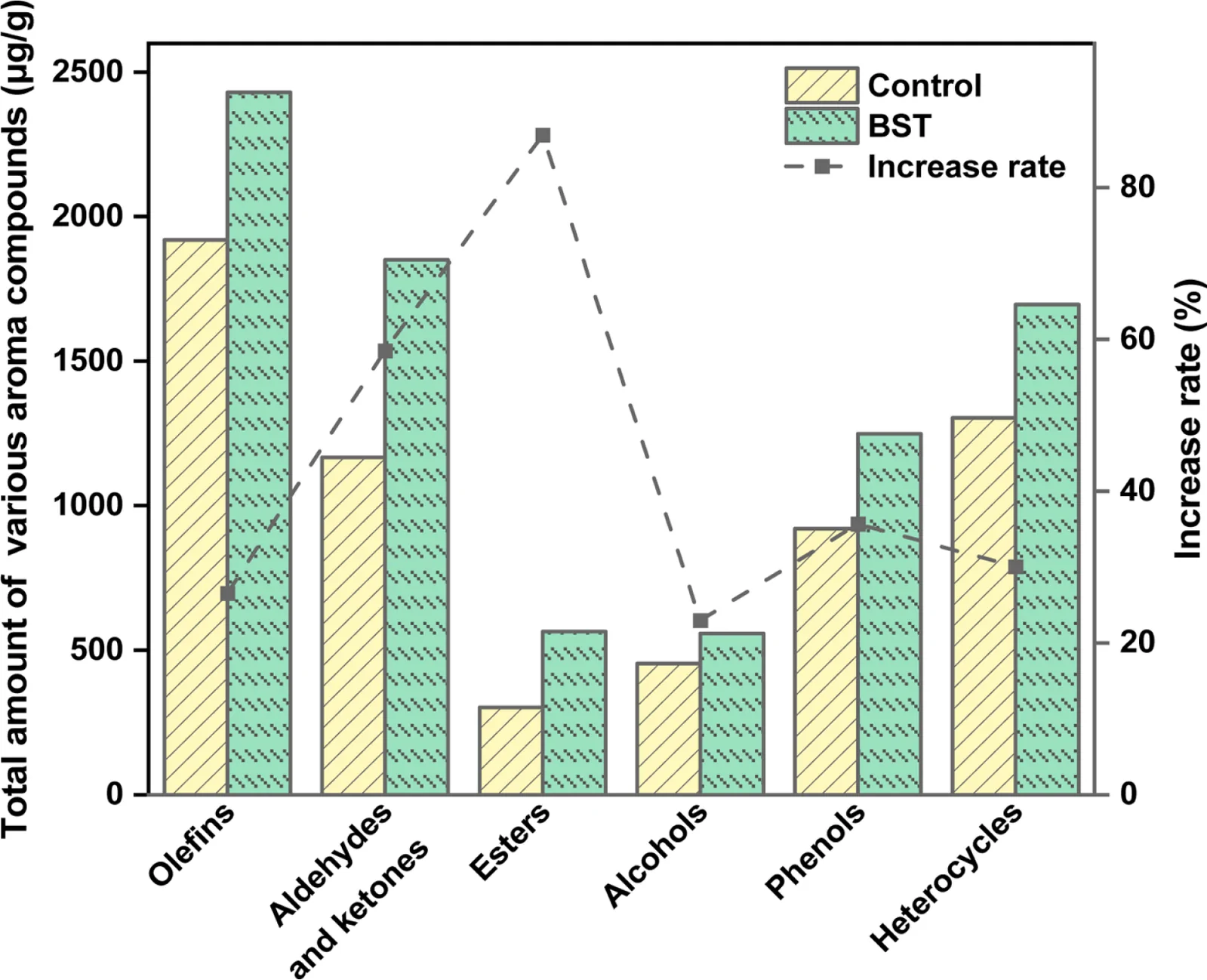
Release profiles of various aroma components in the pyrolysis of cigar tobacco powder. Control: control group; BST: *Bacillus subtilis* DES-59 group.

Py-GC/MS analysis was performed at 350°C to compare the pyrolytic aroma release characteristics of cigar tobacco powder with and without *B. subtilis* DES-59 fermentation. This heating temperature was selected to simulate the typical 300-350°C working temperature of commercial HnB products, enabling an objective evaluation of aroma emission under actual application conditions. The total abundance of major aroma compound categories, including olefins, aldehydes and ketones, esters, alcohols, phenols, and heterocyclics, was higher in the BST group than in the control group, demonstrating that inoculated fermentation effectively enhanced the overall aroma profile of cigar tobacco (Fig. 2). Among these aroma classes, esters exhibited the most remarkable increase (+ 86.95%), followed by aldehydes and ketones (+ 58.53%) and phenols (+ 35.69%) (Fig. 2). The improved aroma release in the BST group may be correlated with proteolytic potential of *B. subtilis* DES-59, which may drive the breakdown of macromolecular proteins to yield free amino acids. Meanwhile, fermentation also elevated the contents of reducing sugars and total soluble sugars (Fig. 1). As crucial Maillard reaction precursors, the accumulated amino acids and sugars jointly facilitated the formation of abundant volatile aroma compounds such as aldehydes, ketones, pyrazines and furans [14, 44].

Representative aroma constituents and compounds with an increase rate above 50% are summarized in Table 1. Several key flavor substances, including trans-cinnamaldehyde, 4,7,9-megastigmatrien-3-one, methyl 2,6-dihydroxybenzoate, β-ionol, 2,6-lutidine, methyl p-hydroxyphenylpropionate, 2,3-dihydrobenzofuran, and 2,4-dimethylbenzoquinoline, increased by more than 100%. Neophytadiene, the most predominant endogenous aroma compound in tobacco, increased by 25.84% in the BST group. As a major degradation product of chlorophyll, neophytadiene possesses a fresh grassy note that offsets undesirable green off-flavors. It can be further degraded into mellow small-molecule volatiles such as furans during fermentation, and also acts as an effective carrier for flavor substances in smoke aerosols, closely correlating with the overall sensory performance of tobacco products [8, 45]. Moreover, *B. subtilis* DES-59 fermentation accelerated carotenoid degradation and the accumulation of their derived volatile products. The carotenoid degradation products 4,7,9-megastigmatrien-3-one and its precursor 9-hydroxy-4,7-megastigmadien-3-one were elevated by 167.62% and 92.26%, respectively. These metabolites contribute typical floral and woody notes [46], further enriching the flavor complexity and coordination of cigar-flavored HnB smoke.

In summary, inoculation with *B. subtilis* DES-59 effectively optimized the pyrolytic aroma profile of cigar tobacco under simulated HnB heating conditions. Remodeling the profile of flavor precursors facilitated the accumulation of key volatile compounds, thereby providing a solid material basis for optimizing the flavor profile and sensory performance of cigar-flavored HnB products.

### Sensory Evaluation of Cigar-Flavored HnB Cigarettes

**Fig. 3:**
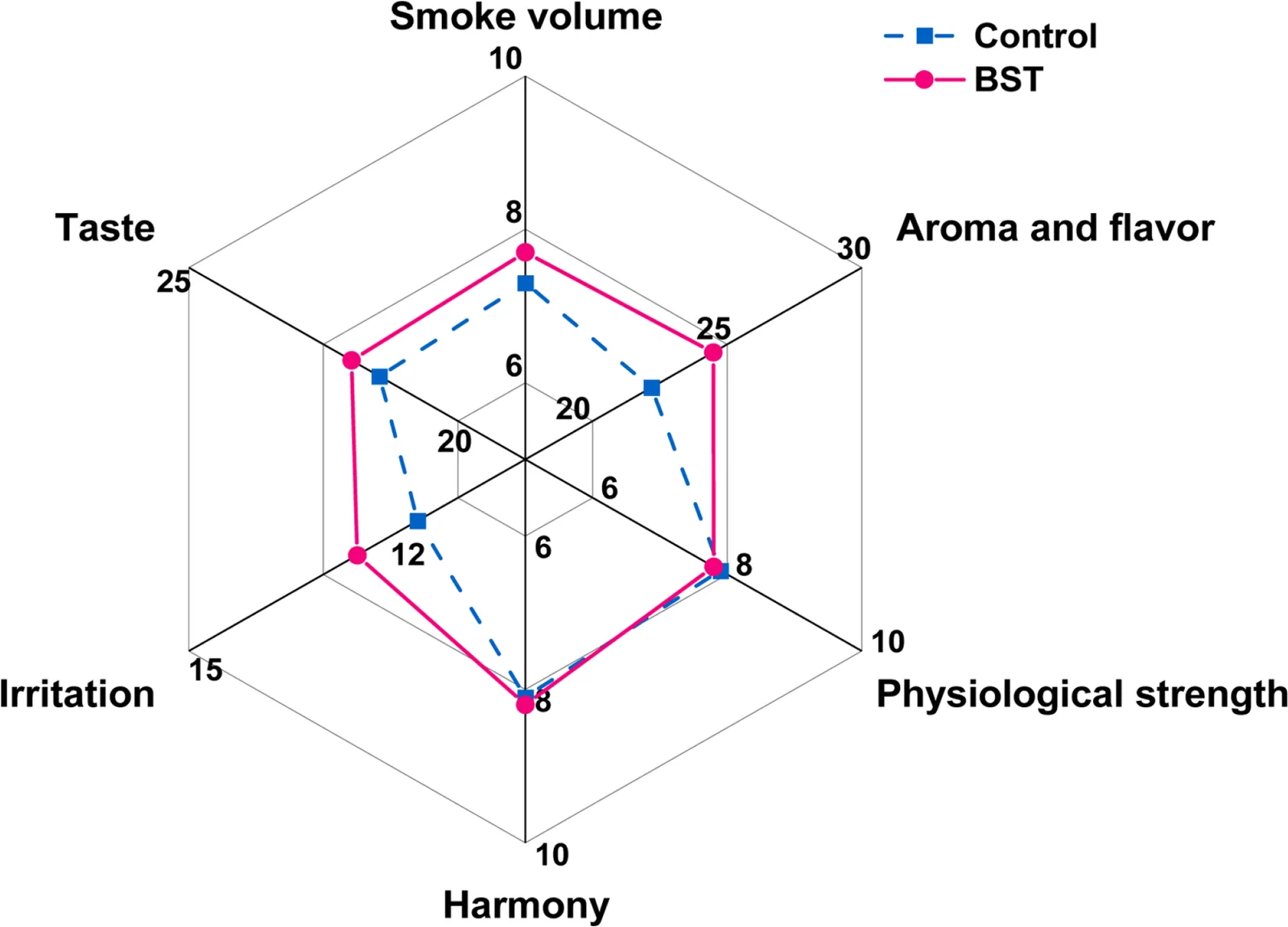
Radar chart of sensory evaluation scores for HnB cigarettes prepared from fermented cigar tobacco powder. Control: control group; BST: *Bacillus subtilis* DES-59 group.

Sensory evaluation was performed in a blind evaluation and random order to eliminate subjective bias from panelists, aiming to clarify the influence of *B. subtilis* DES-59 fermentation on the comprehensive sensory quality of HnB cigarettes supplemented with fermented cigar tobacco powder. Compared with the control group, the total sensory score of the BST group increased from 79.00 to 83.00 (Table S4), accompanied by a more balanced and coordinated distribution in the sensory radar chart (Fig. 3), which demonstrated an obvious improvement in overall sensory performance. Among all evaluation indices, aroma and flavor showed the most prominent enhancement, with the score rising from 22.50 to 24.70 (Table S4). This improvement was highly consistent with the increased abundance of volatile aroma compounds identified via Py-GC/MS analysis (Fig. 2, Table 1). Meanwhile, the irritation score increased from 11.60 to 12.50; slight score elevations were also observed in taste and smoke volume, while harmony and physiological strength remained relatively stable between the two groups (Table S4).

Cigar tobacco is rich in cellulose and protein, which usually produce pungent, bitter, and irritating substances during high-temperature pyrolysis and consequently deteriorate smoking comfort [14, 24]. The reduced irritation and improved taste in the BST group could be reasonably attributed to the effective degradation of protein and cellulose (Fig. 1). In addition, the accumulation of total soluble sugars and reducing sugars further endowed smoke with a mellower and sweeter mouthfeel (Fig. 1) [47]. These soluble sugars also acted as essential Maillard reaction precursors, synergistically promoting the formation of flavor volatiles and further optimizing the overall aroma profile of HnB smoke [47, 48]. Although physiological strength presented a slight downward trend corresponding to the moderate reduction of total alkaloids (Fig. 1), such subtle variation did not exert any adverse impact on the overall sensory experience.

Overall, fermentation with *B. subtilis* DES-59 remarkably improved the sensory quality of cigar-flavored HnB cigarettes. Specifically, it enhanced aroma abundance and flavor richness, mitigated smoke irritation, and maintained the inherent harmony and physiological strength characteristics of cigar tobacco, achieving an overall optimization of sensory performance.

### Microbial Diversity in Fermented Cigar Tobacco Powder

**Fig. 4:**
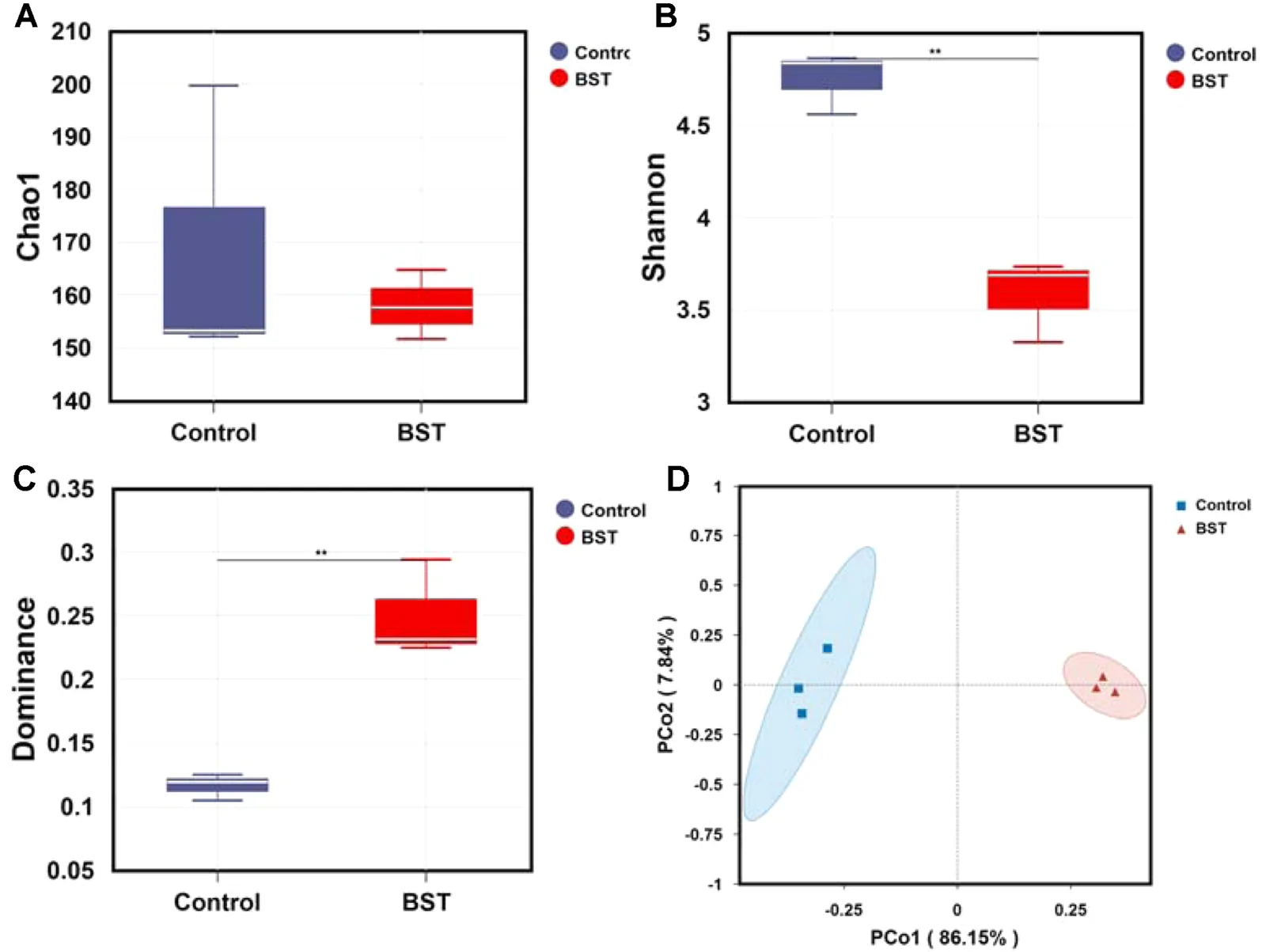
Analysis of microbial diversity in fermented cigar tobacco powder. (**A**) Chao1 index; (**B**) Shannon index; (**C**) Dominance index; (**D**) Principal coordinate analysis (PCoA); Control: control group; BST: *Bacillus subtilis* DES-59 group; Data are presented as mean value ± standard deviation (SD, n = 3). Intergroup differences were analyzed by Duncan’s multiple range test (** *p* < 0.01).

Microbial community diversity profiling was conducted to clarify the regulatory effects of *Bacillus subtilis* DES-59 inoculation on fermented cigar tobacco powder. Alpha diversity results showed that the BST group possessed similar Chao1 index to the control, whereas its Shannon diversity index was significantly lower (*p* < 0.01, Fig. 4A and 4B). The unchanged Chao1 index indicated that exogenous inoculation barely altered the total number of microbial species in tobacco samples. As Shannon index comprehensively reflects species richness and community evenness, its notable decline suggested reduced community uniformity after inoculation. Meanwhile, the significantly elevated Dominance index in the BST group further verified the intensified proliferation advantage of dominant microorganisms (Fig. 4C). Bray-Curtis-based PCoA revealed distinct separation between control and BST samples (Fig. 4D), with PCo1 and PCo2 explaining 86.15% and 7.84% of total community variation, respectively. The high explanatory proportion of PCoA1 demonstrated that microbial community structure was the primary differential factor between two groups. The clear intra-group clustering and inter-group separation pattern confirmed that *B. subtilis* DES-59 inoculation profoundly reconstructed the indigenous microbial ecosystem of cigar tobacco during fermentation.

### Microbial Community Structure and Biomarkers in Fermented Cigar Tobacco Powder

**Fig. 5:**
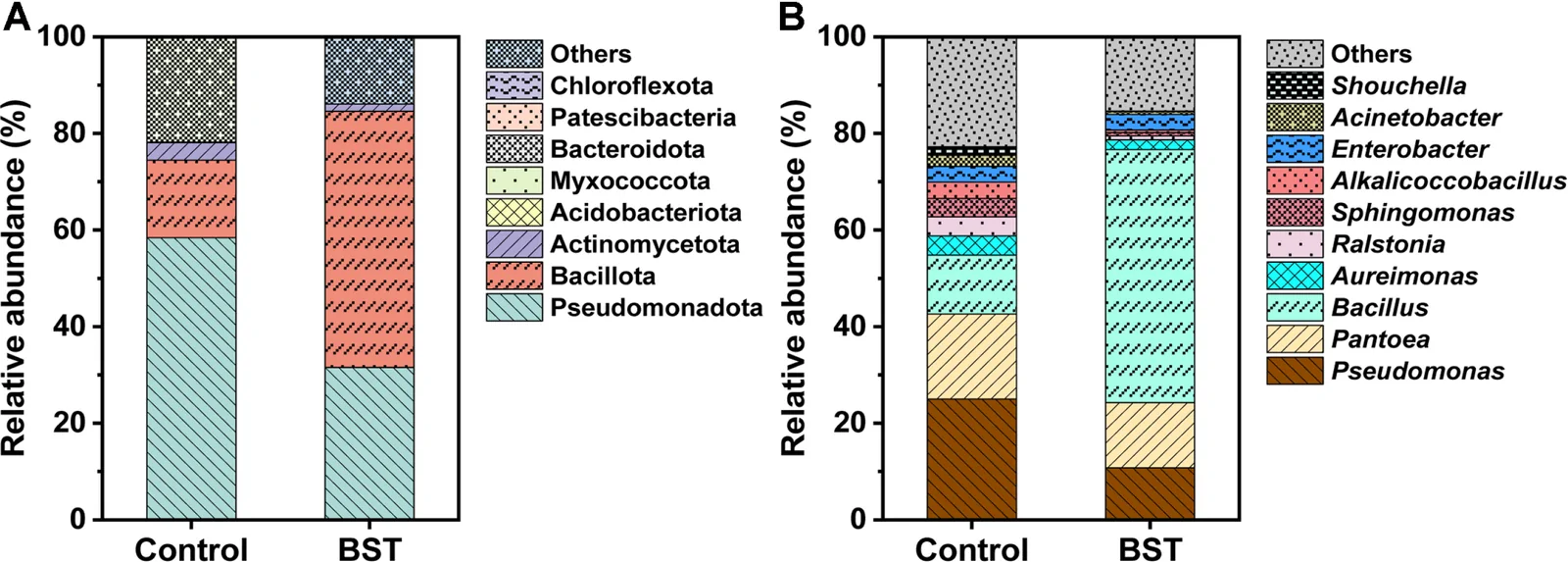
Analysis of microbial community structure in fermented cigar tobacco powder. (**A**) Phylum level; (**B**) Genus level; Control: control group; BST: *Bacillus subtilis* DES-59 group.

**Fig. 6:**
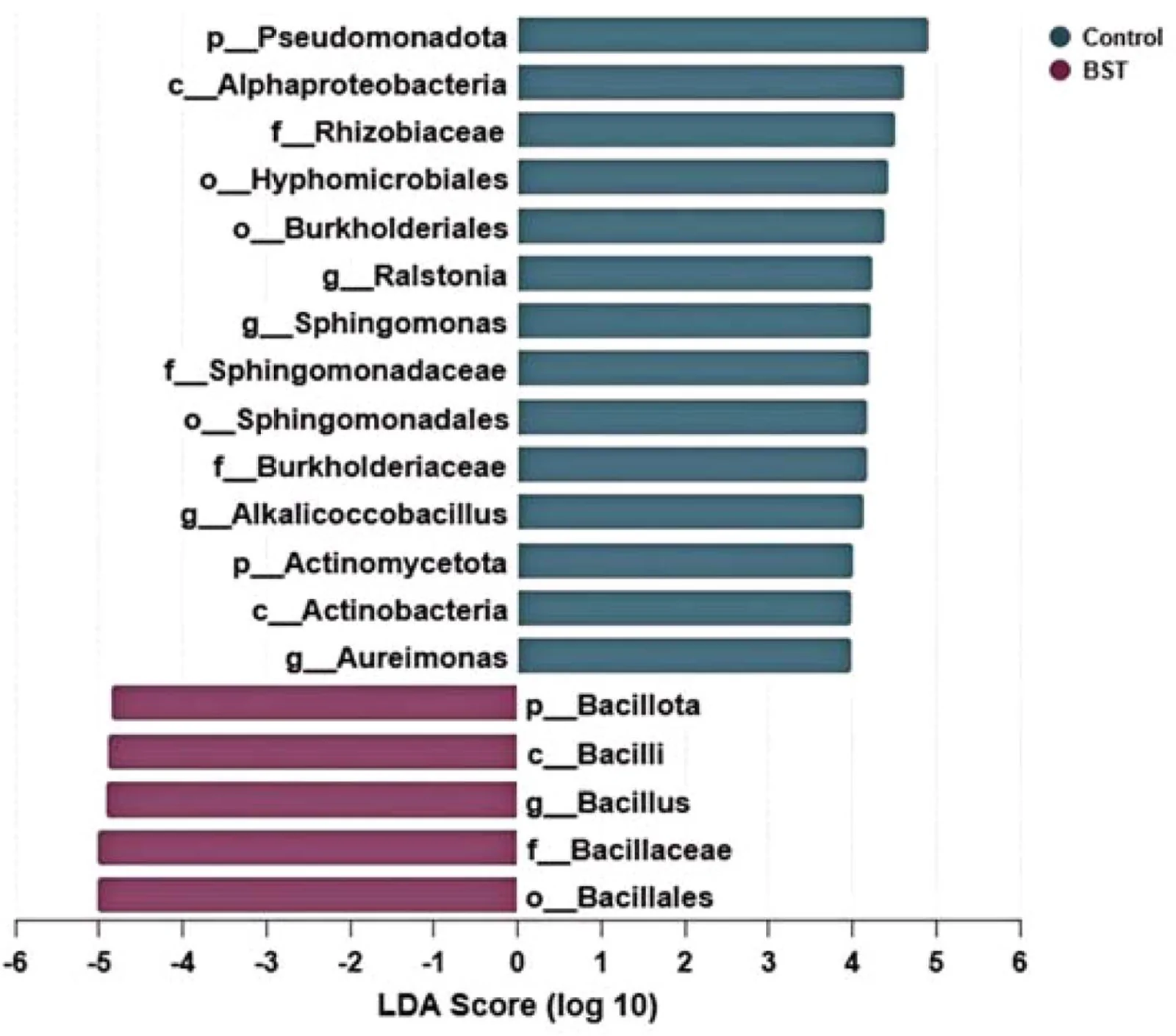
Analysis of microbial biomarkers in fermented cigar tobacco powder. Control: control group; BST: *Bacillus subtilis* DES-59 group.

To further explore the effects of *B. subtilis* DES-59 augmented fermentation on the microbial community structure of cigar tobacco, variations in community composition were analyzed at the phylum and genus levels (Fig. 5). Moreover, linear discriminant analysis effect size (LEfSe) was conducted to identify taxa with significant differential abundance between the control and BST groups (Fig. 6).

At the phylum level (Fig. 5A), Pseudomonadota dominated the control community with a relative abundance of 58.43%, followed by Bacillota accounting for 16.01%. In contrast, Bacillota abundance in the BST group markedly rose to 53.00% and became the predominant phylum, reflecting strong ecological competitiveness of inoculated *B. subtilis*. LEfSe analysis further verified such community shift, as characteristic biomarkers in control samples mainly belonged to Pseudomonadota, including Alphaproteobacteria, Burkholderiales, Sphingomonadales, *Ralstonia*, and *Sphingomonas*, while all differential enriched taxa in BST group belonged to Bacillota, covering Bacilli, Bacillales, Bacillaceae, and *Bacillus*. This typical phylum level community replacement was consistent with previous reports that *Bacillus* strains could drive microbial structure remodeling in fermented tobacco matrices [25].

At the genus level (Fig. 5B), the control group was dominated by three genera: *Pseudomonas* (25.0%), *Pantoea* (17.6%), and *Bacillus* (12.2%). In the BST group, the relative abundance of *Bacillus* sharply increased to 52.4%, becoming the dominant genus. LEfSe analysis confirmed *Bacillus* as the exclusive genus-level biomarker in BST group, verifying its core functional status throughout cigar fermentation. Meanwhile, the abundances of indigenous genera including *Pseudomonas*, *Pantoea*, *Aureimonas*, *Ralstonia*, *Sphingomonas*, *Alkalicoccobacillus*, *Acinetobacter*, and *Shouchella* decreased, suggesting broad niche competition and antagonistic effects exerted by exogenous strains. Existing mechanistic studies have confirmed that *B. subtilis* synthesizes non-ribosomal peptides and polyketides to inhibit heterogeneous microorganisms, reasonably explaining the decline of indigenous bacterial taxa[49]. Notably, *Pseudomonas* and *Pantoea* still maintained relatively high abundance in BST group, indicating stable symbiotic relationships with *B. subtilis* DES-59 and potential synergistic functions in tobacco component transformation. Both genera have been widely reported as crucial flavor-related microorganisms in tobacco fermentation. Fang *et al*. reported a positive correlation between *Pantoea* and characteristic volatiles such as phenylacetaldehyde and acetophenone [50], while Huang *et al*. demonstrated that *Pseudomonas* participated in the formation of flavor-active aldehydes and ketones [25].

In summary, inoculated *B. subtilis* DES-59 reshaped the microbial community structure in cigar tobacco powder, driving the dominant phylum shift from Pseudomonadota to Bacillota, and replacing native dominant genera *Pseudomonas* and *Pantoea* with *Bacillus*. Symbiotic indigenous taxa cooperated with dominant strains to jointly participate in flavor metabolism.

### Prediction of KEGG Metabolic Pathways for Microbial Communities in Fermented Cigar Tobacco Powder

PICRUSt analysis was performed to predict the metabolic functional potential of the microbial community, with a focus on comparing the relative abundances of the top six KEGG pathway categories between the control and BST groups.

**Fig. 7:**
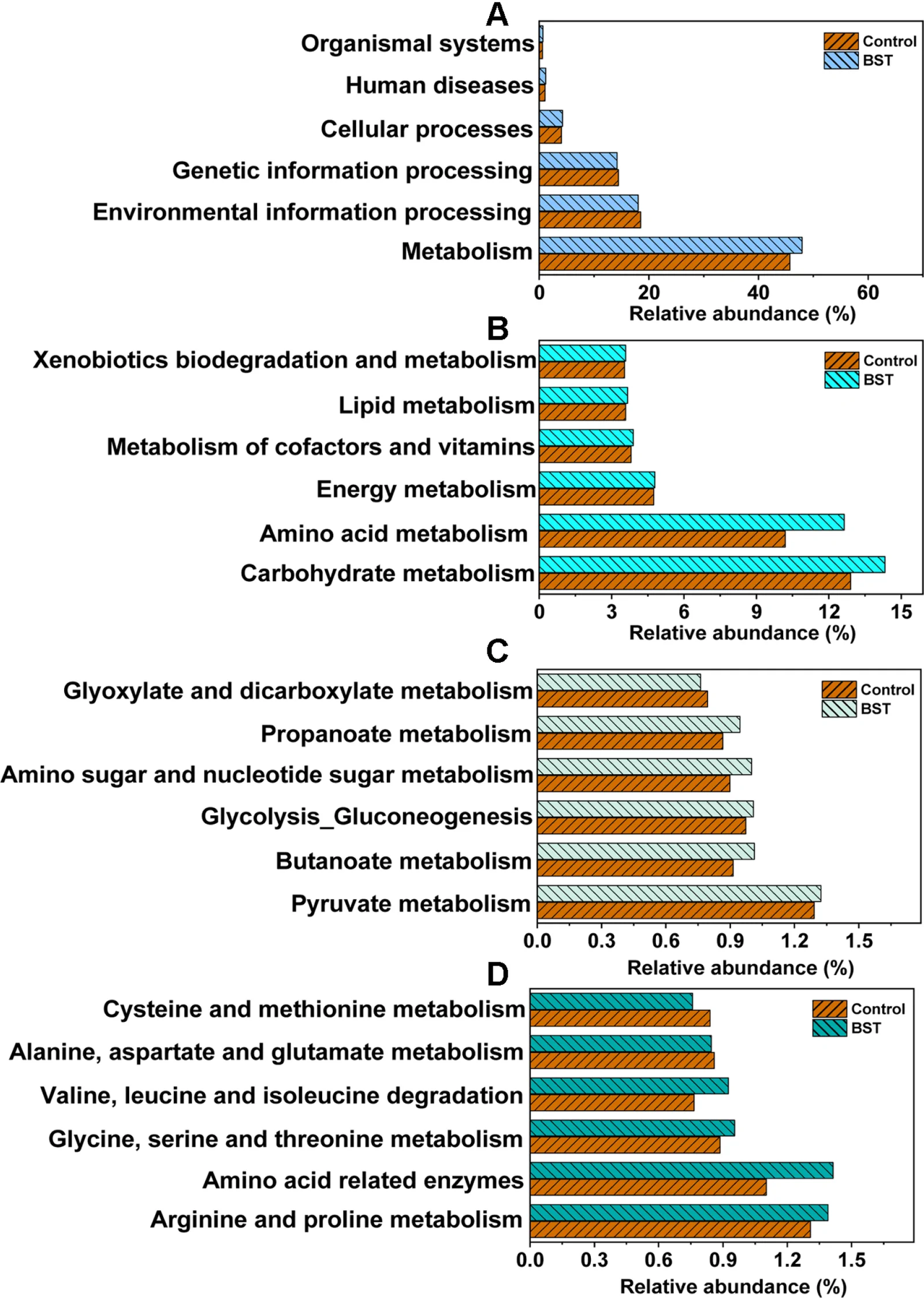
KEGG metabolic pathways prediction of microbial community in fermented cigar tobacco powder. (**A**) Level 1; (**B**) Level 2; (**C**) Level 3 (Carbohydrate metabolism); (**D**) Level 3 (Amino acid metabolism); Control: control group; BST: *Bacillus subtilis* DES-59 group.

At KEGG Level 1, metabolism represented the highest relative abundance in both groups and displayed a higher relative abundance in the BST group (Fig. 7A), suggesting that inoculation with *B. subtilis* DES-59 may potentially enhance overall microbial metabolic activity. At Level 2, carbohydrate metabolism proportion increased from 12.90% to 14.33%, and amino acid metabolism rose from 10.20% to 12.64% in the BST group (Fig. 7B). These functional shifts showed a tentative correlation with chemical composition variations, including a reduction in protein, an increase in amino acids, and a higher concentration of reducing sugars (Fig. 1). At Level 3, carbohydrate metabolism and amino acid metabolism were further analyzed separately. Multiple carbohydrate metabolic pathways were upregulated in the BST group, involving pyruvate metabolism, butanoate metabolism, glycolysis/gluconeogenesis, amino sugar and nucleotide sugar metabolism, and propanoate metabolism (Fig. 7C). The increased predicted potential for carbohydrate metabolism may be associated with the availability of carbon intermediates involved in aroma-related compound formation. As an important glycolytic intermediate, pyruvate can participate in various metabolic pathways leading to the formation of volatile compounds [51], while butanoate and propanoate metabolism were closely associated with ester aroma synthesis [52]. For amino acid metabolism, the BST group showed elevated relative abundance of amino acid related enzymes, as well as increased arginine and proline metabolism, valine, leucine and isoleucine degradation, and glycine, serine and threonine metabolism (Fig. 7D). Arginine and proline can be converted into nitrogen-containing heterocyclic compounds and amine derivatives that contribute to key tobacco smoke aroma [53, 54]. Branched-chain amino acids including valine, leucine and isoleucine can be further converted into precursors responsible for fruity and sweet flavors [55]. Meanwhile, the upregulated glycine, serine and threonine metabolism pathway may promote pyrazine generation through the Maillard reaction, contributing characteristic roasted and nutty notes to the smoke [56].

Overall, these shifts in the relative abundances of KEGG pathways indicate the potential of *B. subtilis* DES-59 fermentation to optimize the aroma profile of cigar tobacco. However, these PICRUSt-based functional predictions only tentatively suggest potential biochemical associations with carbohydrate and amino acid metabolism. Further validation, including targeted metabolite quantification and metatranscriptomic analysis, is required to determine whether the predicted metabolic functions are associated with actual microbial activity and aroma formation.

## Conclusion

In conclusion, this study aimed to address the core challenges of cigar-flavored HnB products, particularly the formation of unpleasant off-flavors and the insufficient aroma compound release under relatively low operating temperatures. For this purpose, the potential of *B. subtilis* DES-59 as a functional strain for cigar tobacco powder optimization was investigated, alongside systematic elucidation of its regulatory effects on chemical composition, pyrolytic aroma, sensory quality and microbial community. Fermentation with *B. subtilis* DES-59 was associated with changes in the chemical composition of cigar tobacco powder, including decreased protein content and increased levels of soluble sugars and free amino acids. These chemical changes were accompanied by an increased release of aroma compounds during thermal pyrolysis at 350°C, with higher relative abundances of esters, aldehydes/ketones, and phenols observed. Representative aroma compounds, including neophytadiene and 4,7,9-megastigmatrien-3-one, also showed increased levels after fermentation. Sensory evaluation indicated improved overall sensory characteristics, particularly in aroma and flavor attributes. Meanwhile, *B. subtilis* DES-59 reshaped the microbial community structure. The dominant microbial population shifted toward Bacillota, and the relative abundance of *Bacillus* increased obviously. Functional prediction based on microbial community data suggested an increased predicted potential for carbohydrate and amino acid metabolism following fermentation. This work demonstrated that *B. subtilis* DES-59 shows promising potential for modulating the quality of cigar tobacco materials. It provides a theoretical basis and technical reference for flavor regulation and industrial application of cigar-flavored heat-not-burn products. Future work will focus on dynamic tracking of fermentation progression, systematic optimization of fermentation parameters, metabolomic validation of microbial metabolic functions, and pilot-scale trials to facilitate the industrial application of *B. subtilis* DES-59 in the production of cigar-flavored HnB products.

## Supplemental Materials

Supplementary data for this paper are available on-line only at http://jmb.or.kr.



## Figures and Tables

**Table 1:** Representative aroma compounds released from the pyrolysis of cigar tobacco powder.

Aroma Compound (μg/g)	Control	BST	Change (%)
Neophytadiene	1753.43	2206.53	+25.84
Benzaldehyde	19.27	28.99	+50.44
trans-Cinnamaldehyde	54.42	152.34	+179.93
9-Hydroxy-4,7-megastigmadien-3-one	123.67	237.77	+92.26
4,7,9-Megastigmatrien-3-one	142.93	382.51	+167.62
3-Ethyl-4-methyl-1H-pyrrole-2,5-dione	67.56	104.71	+54.99
3-Methyl-2-cyclopenten-1-one	16.98	30.65	+80.51
Methyl 2,6-dihydroxybenzoate	31.53	86.32	+173.77
Methyl p-hydroxyphenylpropionate	34.51	184.23	+433.85
β-Ionol	24.16	56.81	+84.95
Phenethyl alcohol	35.48	65.62	+84.95
(3S,5R,8S,7Z,9ζ)-5,6-Epoxy-7-megastigmen-3,9-diol	125.88	198.25	+57.49
2,6-Dimethoxyphenol	57.69	87.59	+51.83
o-Cresol	60.63	119.62	+97.30
2,3-Dihydrobenzofuran	56.43	146.86	+160.25
2,6-Lutidine	28.63	85.69	+199.30
2-Methyl-3-hydroxypyridine	41.68	63.16	+51.54
2,4-Dimethylbenzoquinoline	37.85	153.61	+305.84

Control: control group; BST: *Bacillus subtilis* DES-59 group.
