# Supplementary material for: Bacillus subtilis DES-59 Modulates Microbial Community Structure and Aroma Formation in Cigar Fermentation to Improve the Quality of Heat-Not-Burn (HnB) Tobacco
Source: J Microbiol Biotechnol. 2026 Sep 2;36:e2605012. doi: 10.4014/jmb.2605.05012 (PMC13559716; doi:10.4014/jmb.2605.05012)
Supplement: Supplementary file 1 [file jmb-36-e2605012-supple.pdf]

J. Microbiol. Biotechnol. 2026. 36: e2605012
https://doi.org/10.4014/jmb.2605.05012

Supplementary Tables

Bacillus subtilis DES-59 modulates microbial community structure and aroma formation in cigar fermentation to improve the quality of heat-not-burn (HnB) tobacco

Lu-Xin Liang[1], Ming-Jun Zhu[1,*], and Lu Zhao[2,3,4*]

[1] School of Biology and Biological Engineering, Guangdong Key Laboratory of Fermentation and Enzyme Engineering, South China University of Technology, Guangzhou Higher Education Mega Center, Panyu, Guangzhou 510006, P. R. China
[2] Yunnan Academy of Tobacco Agricultural Sciences, Kunming 650021, P. R. China
[3] China Tobacco Breeding Technology Innovation Center, Kunming 650021, P. R. China
[4] Yunnan Daguan Laboratory, Kunming 650021, China

*Correspondence:

Lu Zhao, zhaolu66@outlook.com; / Ming-Jun Zhu, mjzhu@scut.edu.cn

Table S1. Moisture content of cigar tobacco powder.

Group	Moisture content at Day 0 (%)	Moisture content at Day 7 (%)
Control	30.0 ± 0.2	26.3 ± 0.1
BST	30.0 ± 0.2	25.8 ± 0.2

Control: control group; BST: *Bacillus subtilis* DES-59 group; Data are presented as mean value ± standard deviation (n = 3).

Table S2. Viable bacterial counts at the initial and final stages of fermentation.

Group	Viable bacterial counts at Day 0 (CFU/g)	Viable bacterial counts at Day 7 (CFU/g)
Control	$(3.3 \pm 0.4) \times 10^4$	$(1.4 \pm 0.3) \times 10^5$
BST	$(2.1 \pm 0.3) \times 10^8$	$(1.7 \pm 0.2) \times 10^7$

Control: control group; BST: Bacillus subtilis DES-59 group; Viable bacterial counts were determined via plate counting on LB solid medium. Data are presented as mean ± standard deviation (n = 3).

Table S3. Evaluation indexes and scoring standard of sensory quality of heat-not-burn (HnB) cigarettes.

Sensory index	Evaluation Standard		
	Grade I (Full score)	Grade II (Full score)	Grade III (Full score)
Smoke volume	Abundant smoke (10)	Slightly less smoke (8)	Weak smoke (6)
Aroma and flavor	Full-bodied and smooth aroma (30)	Sufficient aroma with little roughness (25)	Light and rough aroma (20)
Physiological strength	Moderate strength (10)	A slightly strong or a slightly insufficient strength (8)	Excessively strong or insufficient strength (6)
Harmony	Well harmonious (10)	Generally harmonious (8)	Tolerably harmonious (6)
Irritation	Free from Irritation (15)	Slightly irritating (12)	Quite irritating (9)
Taste	Comfortable (25)	Generally Comfortable (22)	Tolerable (20)

Table S4. Sensory evaluation scores for HnB cigarettes prepared from fermented cigar tobacco powder.

Sensory evaluation indicators	Control	BST
Smoke volume	7.30 ± 0.57 a	7.70 ± 0.45 b
Aroma and flavor	22.50 ± 1.23 a	24.70 ± 1.15 b
Physiological strength	7.90 ± 0.22 a	7.80 ± 0.27 a
Harmony	8.10 ± 0.22 a	8.20 ± 0.27 a
Irritation	11.60 ± 0.55 a	12.50 ± 0.50 b
Taste	21.60 ± 0.42 a	22.10 ± 0.65 b
Total sensory score	79.00 ± 1.23 a	83.00 ± 1.70 b

Control: control group; BST: *Bacillus subtilis* DES-59 group; Data are presented as mean value ± standard deviation (SD, n = 3). Different lowercase letters indicate significant differences among groups at $p < 0.05$ according to Duncan’s multiple range test.

Supplementary Method S1. GC/MS analysis of aroma components in pyrolysis smoke

In the GC/MS experiment, an HP-5MS column (Agilent 19091S433, 30 m × 0.25 × 0.25) was employed. The injector temperature was set at 260°C with an injection volume of 10 μL and a split ratio of 20:1. The interface temperature was maintained at 280°C, and the temperature program consisted of two stages: initially ramping from 60°C to 200 °C at a rate of 2°C/min, followed by another ramp to 220°C at the same rate, and finally a ramp to 260°C at 10°C/min with a hold time of 15 min. The flow rate was set at 1.2 mL/min. The ion source temperature was 230°C, and the quadrupole temperature was 150°C. The mass spectrometry scan range was 30-550 amu, and the substance retrieval library used was NIST08.

For metabolite identification via GC-MS, mass spectra were matched against the NIST 08 library. The dot product (forward match factor) and reverse dot product (reverse match factor) were set as the key evaluation indicators, with explicit cutoff values of ≥700 for both. Only metabolites meeting this dual threshold were considered valid annotations to ensure the reliability of the results.

Quantitation: A semi-quantitative method was used. The relative content of each compound was calculated based on the peak area ratio to the internal standard (decanol) using the following formula:

$$C_x = \frac{\mathrm{A}_x}{A_{IS}} \times C_{IS}$$

Where C_x is the calculated concentration of the target compound, A_x is its peak area, A_{IS} is the peak area of the internal standard, and C_{IS} is the concentration of the internal standard.
